# Biological Control of *Bradysia odoriphaga* Using *Coenosia attenuata* with Banker Media System and Mulch Layer

**DOI:** 10.3390/insects17101063

**Published:** 2026-10-15

**Authors:** Deyu Zou, Huihui Wu, Thomas A. Coudron, Yang Liu, Haiyan Ben, Weihong Xu, Jingyang Xu, Yuqiu Cong, Yuxin Bu, Boxuan Hu

**Affiliations:** 1 Institute of Plant Protection, Tianjin Academy of Agricultural Sciences, Tianjin 300384, China; zoudeyu@taas.ac.cn (D.Z.); benhaiyan@taas.ac.cn (H.B.); cyq13314013469@126.com (Y.C.); 2 Mass Production Facility of Natural Enemy Insects of Tianjin Academy of Agricultural Sciences, Key Laboratory of Natural Enemies Insects, Ministry of Agriculture and Rural Affairs, Tianjin 301723, China; 3 College of Horticulture and Landscape, Tianjin Agricultural University, Tianjin 300392, China; wuhuihui@tjau.edu.cn (H.W.); liuyang@tjau.edu.cn (Y.L.); 15731957328@163.com (Y.B.); hubobear@163.com (B.H.); 4 Biological Control of Insects Research Laboratory, USDA-Agricultural Research Service, Columbia, MO 65203-3535, USA; coudront@missouri.edu

**Keywords:** *Allium tuberosum*, banker media system, *Bradysia odoriphaga*, *Coenosia attenuata*, fungus gnat management, mulch layer

## Abstract

Greenhouse cage trials were conducted to evaluate the biocontrol potential of the predatory tiger fly *Coenosia attenuata* against the Chinese chive maggot *Bradysia odoriphaga* infesting *Allium tuberosum*. To ensure that adult *C. attenuata* have sufficient food when the *B. odoriphaga* adults decline or are absent, we supplemented the predator with alternative prey (*Drosophila melanogaster* adults), a banana-based banker medium, and a coir mulch layer across four treatments with three replicates per treatment over 50 weeks. When *C. attenuata* was released alone (T1), corrected control efficacy against *B. odoriphaga* larvae ranged from 56.14% to 59.82%. Notably, peak larval suppression was significantly higher in treatments combining the predator with a mulch layer (T2: 94.23%) or with the banker media system (T4: 100%). Against adult stages, T1 achieved 32.68–69.73% corrected efficacy, whereas T2 and T4 reached peak values of 100% and 97.69%, respectively. Predator application also doubled the total cumulative dry weight of Chinese chive. However, the concurrent use of banker medium and mulch layer (T3) did not produce synergistic effects. Collectively, these findings demonstrate that banker media systems and coir mulch layers are effective auxiliary tools for enhancing *C. attenuata*-based biocontrol in protected cultivation systems.

## Simple Summary

Chinese chive maggot, *Bradysia odoriphaga*, is a soil-dwelling pest of Chinese chives, onions, garlic, and others. It can cause yield losses of 30 to 80% and, in severe cases, crops must be replanted. Chemical insecticides, such as clothianidin and chlorfenapyr, applied as a soil drench, are the main approach for *B. odoriphaga* management in China and elsewhere, raising human health concerns. The predaceous tiger fly, *Coenosia attenuata*, preys upon *B. odoriphaga* adults under laboratory conditions; however, field-based biocontrol of *B. odoriphaga* using *C. attenuata* has not previously been reported. In this study, we established a sustainable management strategy for the Chinese chive maggot by deploying *C. attenuata* in combination with a banker media system and a mulch layer. Our results indicate that corrected control efficacy against *B. odoriphaga* larvae and adults is significantly greater in treatments incorporating coir mulch or the banker media system than in control treatments lacking these components. Furthermore, this sustainable strategy nearly doubled the dry weight of Chinese chives under greenhouse conditions, with performance comparable to plants treated with entomopathogenic nematodes and imidacloprid.

## 1. Introduction

The Chinese chive maggot, *Bradysia odoriphaga*, infests more than 30 economically important crops belonging to at least seven plant families, such as Chinese chives, onions, garlic, ginger, and others [1,2]. The soil-dwelling larvae of this fungus gnat feed on these crop roots and stems and can lead to chive production losses of 30% to 80% in North China [3]. In the absence of insecticide treatments, maggot damage can destroy an entire crop [4,5]. Chemical insecticide application is the main approach for *B. odoriphaga* management in China and elsewhere [6]. Repeated application of chemical insecticides has led to rapid development of insecticide resistance in *B. odoriphaga* populations and has resulted in environmental pollution and high insecticide residues on marketed vegetables [7,8].

Entomopathogenic nematodes (EPNs) have been reported as potential biological control agents for several species in the genus *Bradysia*. *Heterorhabditis* sp. was shown to be effective against *B. odoriphaga* [9,10]. Ma et al. 2013 [5] evaluated the virulence of 145 isolates from 13 species of EPNs against *B. odoriphaga* and found a *Steinernema feltiae* isolate to perform best in field applications. Wu et al. 2015 [11] evaluated combinations of EPNs and sticky traps and found that an *S. feltiae* isolate in combination with black sticky traps performed best against third-instar *B. odoriphaga* larvae. Despite their demonstrated potential, EPNs have seen limited application against *B. odoriphaga* due to their vulnerability to soil conditions. Likewise, thermal control methods, though effective at temperatures exceeding 37 °C for 4 h [12,13], are constrained by practical complexities. Some chemosensory genes and proteins, such as BodoCSP4, *BodoCSP6* and *BodoCSP7*, play crucial roles in host identification of *B. odoriphaga* [14]. However, there is still a long way to go before these genes and their encoded proteins can be applied for pest control [15]. In contrast, the exploration of natural enemy insects for controlling this pest has received little attention. In greenhouses overseas, soil predatory mites such as *Stratiolaelaps scimitus*, together with rove beetle *Dalotia coriaria*, have been commercialized for the management of *Bradysia* fungus gnats [16,17]. However, these studies mainly focused on greenhouse sciarid species other than *B. odoriphaga*. Up to now, the application of natural enemy insects for managing adults of *B. odoriphaga* has not been documented.

The predaceous fly *Coenosia attenuata* is currently thought to be a useful biological pest control agent in greenhouses. It is also known as “tiger fly”, “killer fly”, or “hunter fly” [18,19,20,21]. It has been extensively studied because of its ability to effectively suppress a wide range of agricultural pests, such as fungus gnats, whiteflies, leaf miners, winged aphids, leafhoppers, midges, moth flies, shore flies, and fruit flies [22,23,24,25,26,27,28,29]. A wide range of prey used as food makes them very flexible; hence, especially useful. *C. attenuata* adults live above the soil and feed on flying insects, but its larvae are soil-dwelling and feed on small, soft-bodied soil invertebrates like the larvae of sciarids and shore flies. *C. attenuata* is native to Europe and was likely introduced via plant material. It has since become distributed worldwide and has been documented in several regions of China, including Taiwan, Xinjiang, Yunnan, and Tianjin [29,30,31,32,33,34].

Our previous research demonstrated that each adult *C. attenuata* was capable of preying on approximately 18.5 to 24 adult *B. odoriphaga* per day under laboratory conditions [33,35], highlighting its potential as an effective biological control agent against *B. odoriphaga*. *Coenosia attenuata* adults can develop through their life cycle by preying on *Drosophila melanogaster* adults, a species that does not damage Chinese chives. Following emergence of the next generation of *B. odoriphaga* adults, removing the banana banker medium and alternative prey (*D. melanogaster*) enables *C. attenuata* adults to switch to preying on *B. odoriphaga* adults. These features reveal the potential of *C. attenuata* for use in banker systems. In this study, *C. attenuata* was used for the biological control of *B. odoriphaga* infesting Chinese chive (*Allium tuberosum*) in the greenhouse. *Drosophila melanogaster* adults (an alternative prey), bananas (a banker medium), and coir substrate (a mulch layer) were applied to ensure that adult *C. attenuata* have sufficient food when *B. odoriphaga* adults decline or are absent. We hypothesize that the addition of a mulch layer and/or a banker media system can improve the persistence of *C. attenuata* and consequently increase its suppression of *B. odoriphaga*.

## 2. Materials and Methods

### 2.1. Experimental Bradysia odoriphaga

Approximately 200 *B. odoriphaga* adults were collected from a greenhouse at the Institute of Plant Protection of Tianjin Academy of Agricultural Sciences (Xiqing District, Tianjin, China), and 20 to 30 pairs of newly emerged *B. odoriphaga* adults were placed in transparent plastic rearing containers (8.5 cm top diameter, 11.5 cm bottom diameter, and 6 cm high). The top of the plastic pot was modified to include one 3.0 cm diameter hole covered with fine screen (0.4 mm mesh openings) for ventilation. A Petri dish (11 cm diameter) served as the bottom. A piece of Chinese chive bulb (4 mm thick), serving as an oviposition site, was placed on a piece of filter paper in the Petri dish containing a layer of 2.5% water agar (Beijing Aoboxing Biotech Co., Ltd., Beijing, China). Adult females oviposited for a period of 2 to 4 days and died. About 3 days later, larvae hatched, and Chinese chive stem segments were placed on the Petri dish as food. The duration of larvae and pupae was about 25 days and 5 days, respectively. The colony was maintained in a laboratory incubator (Ningbo Jiangnan Instrument Factory, Ningbo, China) and held at 24 ± 1 °C, 14:10 (L:D), and 70 ± 5% RH. The laboratory colony of *B. odoriphaga* was maintained for four generations before being used in the experiment.

### 2.2. Experimental Bradysia impatiens

The larvae of *B. impatiens* were used as prey for larvae of *C. attenuata* because of their shorter development time. A colony of fungus gnats was initiated with about 100 *B. impatiens* adults collected from a greenhouse at the Modern Agricultural Science and Technology Innovation Base (Wuqing District, Tianjin, China). *Bradysia impatiens* were reared using a simplified method reported by Zou et al. 2021 [33]. Briefly, 300 mL of black peat (Lvdimeijing Science and Technology Co., Ltd., Beijing, China) and 55 to 60 g of dry kidney bean powder (Muyi Grain and Oil Sales Center, Tianjin, China) were placed in an open plastic box (25.5 cm × 19 cm × 7.8 cm). The mix was then moistened with 250 mL of tap water, and a 0.2 cm thick layer of moist coir (Shanghai Galuku Agricultural Science and Technology Co., Ltd., Shanghai, China; desalted, EC = 0.5, family pack, common grade) was placed on the top of the mix. Then the open plastic box was placed in a tissue bag (50 cm × 35 cm, 0.04 cm mesh openings). Four hundred to 500 newly emerged adult *B. impatiens* were placed in the tissue bag and closed with a binder clip. Fresh rearing medium was prepared daily, and new cultures were set up daily. The colony was maintained in a laboratory incubator and held at 24 ± 1 °C, 14:10 (L:D), and 70 ± 5% RH.

### 2.3. Experimental Coenosia attenuata

The *C. attenuata* used to establish a laboratory colony for this study were collected at Leizhuangzi flower farm in Tianjin, China. Adults were introduced to a cage (50 cm × 70 cm × 50 cm), containing oviposition media consisting of an open plastic box (20 cm × 29 cm × 7 cm) with eggs of *B. impatiens* on rearing media as described above, and 1 cm thick layer of moist coir placed on top of the rearing media for oviposition. *Drosophila melanogaster* adults were supplied as prey every day to adult *C. attenuata*. Five to 6 days later, the oviposition media were replaced with a new one. The exposed oviposition media were transferred to another cage, and second- and third-instar larvae of *B. impatiens* were added to the box to feed larvae of *C. attenuata*. Distilled water (Yongqingyuan Distilled Water Business Department, Tianjin, China) was added to the box when the media became dry. About 20 to 21 days later, adults of *C. attenuata* emerged. The colony was maintained in a laboratory incubator and held at 25 ± 1 °C, 14:10 (L:D), and 70 ± 5% RH.

### 2.4. Biological Control of B. odoriphaga Using C. attenuata in Greenhouse Cages

Fifteen nylon mesh cages (1 m × 1.5 m × 1 m, 0.0165 cm mesh openings) were arranged in a greenhouse at the Modern Agricultural Science and Technology Innovation Base (Wuqing District, Tianjin, China). Each cage was fully enclosed on six sides, with an 80 cm long zipper installed on one side to serve as an access door. Twenty healthy 1-year-old Chinese chive plants were transplanted into each plastic flowerpot (21 cm top diameter, 13.5 cm bottom diameter, and 20 cm high) filled with a sterilized substrate containing equal volumes of black peat and peat moss. Four grams of compound fertilizer (N:P:K = 5:2:1) (Sinopharm Chemical Reagent Co., Ltd., Shanghai, China) were added to each pot. Ten labeled flowerpots (numbered 1–10) were placed inside each cage and arranged in two rows of 5 pots. One blank control CK and 4 treatments (denoted T1–T4) were tested in this study; the treatments comprised different combinations of maggots, tiger flies, mulch layers, and a banker media system (containing banker media (bananas) and alternative prey (*Drosophila* flies)), as detailed in Table 1. We performed intermittent releases when the natural enemy population declined. This approach aligned with conventional farming practices. In addition, we supplied alternative prey to maintain the ratio of natural enemies to prey (including *B. odoriphaga* and *D. melanogaster*) at approximately 1:6–15. Each treatment was replicated 3 times (3 cages). Climate data were recorded using a HOBO data logger, model U23-001 (Onset Computer Corporation, Bourne, MA, USA).

The experiment was initiated on 6 May 2025. Adults of *B. odoriphaga* and *C. attenuata* in each cage were counted weekly using a hand-held counter (Wenzhou Yika Electric Co., Ltd., Wenzhou, China), while larvae were enumerated from the soil of differently located flowerpots bearing identical numbers. Following larval counting, Chinese chive plants, soil, and larvae were returned to their original flowerpots. The corrected control efficacies against *B. odoriphaga* were evaluated at 3, 10, 20, 30, 40, and 50 weeks after the start of the experiment. Dead Chinese chive plants were replaced with healthy individuals if all 20 chive plants within a pot perished. The exact number of replaced plants was not recorded; however, replacements occurred at a low frequency, and these substituted plants were excluded from all plant growth and yield analyses to avoid bias. All chives were mown above ground at the start of the experiment. Dry weight (g), samples were dried at 90 °C for 9 h using a laboratory oven (Model UFE 600, Memmert GmbH + Co. KG, Schwabach, Germany); measured for all ten flowerpots in each cage, *n* = 3) was measured every four weeks. Plant height (cm) and chlorophyll content (SPAD value; measured with an SPAD-502 chlorophyll meter, Konica Minolta Sensing, Inc., Osaka, Japan; readings taken on the middle leaf portion of the ten tallest plants per cage) were measured every four weeks on the ten tallest plants subsampled from each cage. Mean values per cage were used for statistical analysis (*n* = 3 independent cages per treatment).

### 2.5. Statistical Analyses

Abbott-corrected control efficacy was arcsine square-root transformed prior to statistical analysis. Shapiro–Wilk test was used to examine the normality of residuals, and Levene’s test was used to assess homogeneity of variances. Comparisons of plant height, chlorophyll content, dry weight, and corrected control efficacy were conducted using one-way ANOVA with Tukey’s HSD test (α = 0.05) under homogeneous variances; Welch’s ANOVA and the Games–Howell test (α = 0.05) were used when variances were heterogeneous. All the statistical tests were done using SAS version 9.4. The corrected control efficacy was calculated using the Abbott formula [36].

## 3. Results

### 3.1. Corrected Control Efficacy Against B. odoriphaga Larvae in Greenhouse Conditions with Chinese Chive

**Figure 1:**
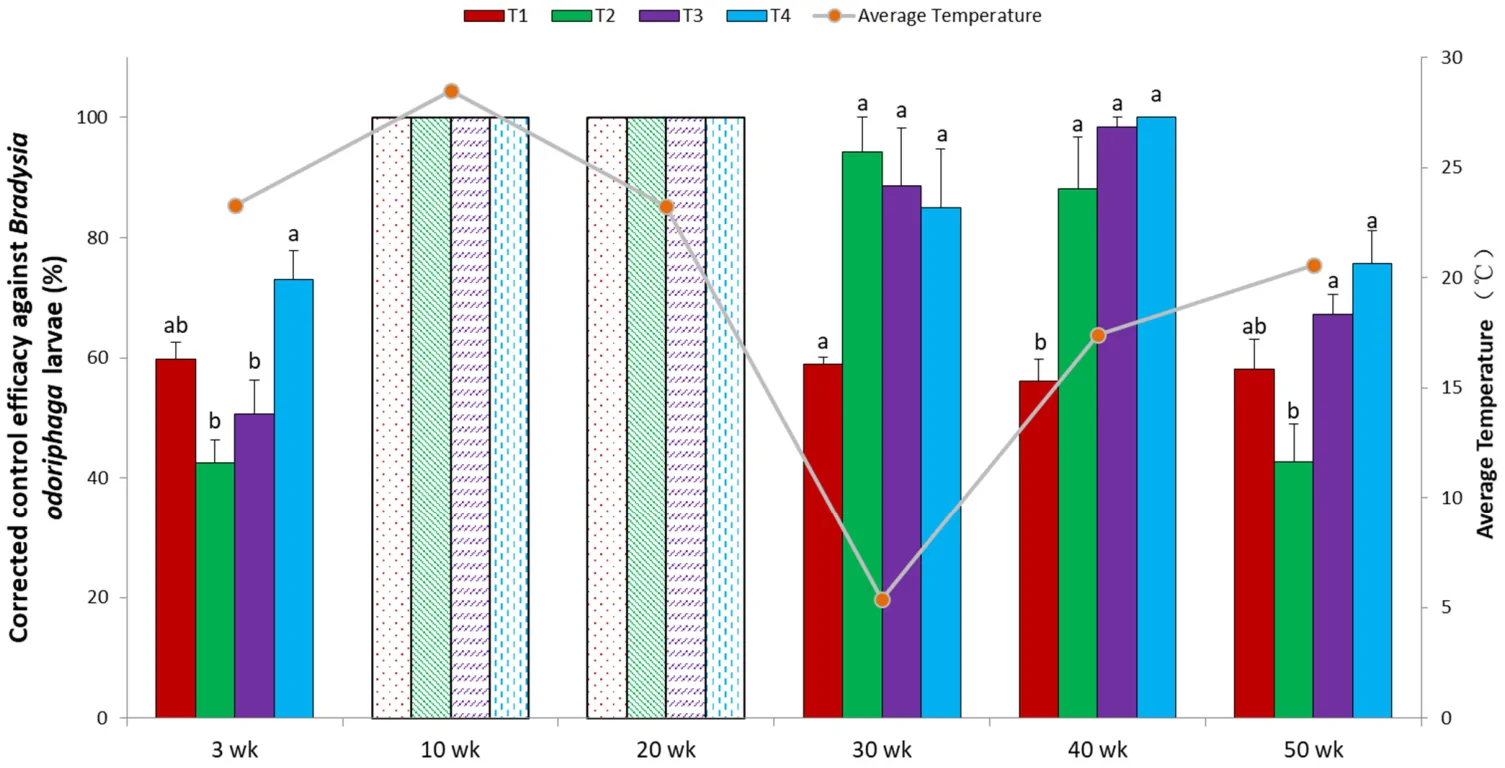
Corrected control efficacy against *Bradysia odoriphaga* larvae under greenhouse conditions in different treatments. Values are mean ± SE. Different letters above columns indicate significant differences based on one-way ANOVA followed by Tukey’s HSD test; *p* < 0.05. CK, no control measure; T1, *Coenosia attenuata* alone; T2, *C. attenuata* + mulch layer; T3, *C. attenuata* + mulch layer + banker media system; T4, *C. attenuata* + banker media system. At weeks 10 and 20, high-temperature stress caused complete mortality of pests in both the control and all treatment groups. Corrected control efficacy could not be calculated. Raw mortality reached 100% at these two time points, which was not Abbott-corrected.

The corrected control efficacy against *B. odoriphaga* larvae with different treatments was shown in Figure 1. The fungus gnat larvae were suppressed by *C. attenuata* larvae during the subsequent 50 weeks, with the corrected control efficacy of 56.14% at 40 weeks and 59.82% at 3 weeks in T1. The corrected control efficacy against *B. odoriphaga* larvae was significantly higher for T4 than T2 and T3 at 3 weeks (*F*_3,8_ = 8.84, *p* = 0.0064). At weeks 10 and 20, high-temperature stress caused complete mortality of pests in both the control and all treatment groups. Corrected control efficacy could not be calculated. Raw mortality reached 100% at these two time points, which was not Abbott-corrected.

The fungus gnat larvae were suppressed by *C. attenuata* larvae during the subsequent 50 weeks, with the corrected control efficacy of 42.47% at 3 weeks to 94.23% at 30 weeks in T2 (Figure 1). There was no significant difference in corrected control efficacy in T1, T2, T3, and T4 at 30 weeks (*F*_3,8_ = 3.55, *p* = 0.0673), although the corrected control efficacy against *B. odoriphaga* larvae in T1 was 26.1% to 35.29% lower than the other three treatments. At 40 weeks, there was no significant difference in corrected control efficacy in T2, T3, and T4, but all of them were significantly higher than that of T1 (*F*_3,8_ = 15.88, *p* = 0.001). At 50 weeks, corrected control efficacy against *B. odoriphaga* larvae did not differ significantly between T1 and T2. However, T3 and T4, which utilized banker media, exhibited significantly higher corrected control efficacy than T2 (*F*_3,8_ = 7.46, *p* = 0.0105).

In treatment T3 with banker media and a mulch layer, *C. attenuata* larvae suppressed fungus gnat larvae over the subsequent 50 weeks, with corrected control efficacy varying from 50.68% at 3 weeks to 98.46% at 40 weeks (Figure 1). However, treatment T3 exhibited significantly higher corrected control efficacy than that of T2 (*F*_3,8_ = 7.46, *p* = 0.0105) but only at 50 weeks. In treatment T4 containing the banker media system, *C. attenuata* larvae suppressed fungus gnat larvae across the subsequent 50-week period, with corrected control efficacy ranging from 73.03% at 3 weeks to 100% at 40 weeks (Figure 1). Treatment T4 exhibited significantly higher corrected control efficacy than treatment T2 (mulch-layer treatment) at both 3 weeks and 50 weeks.

### 3.2. Corrected Control Efficacy Against B. odoriphaga Adults in Greenhouse Conditions with Chinese Chive

**Figure 2:**
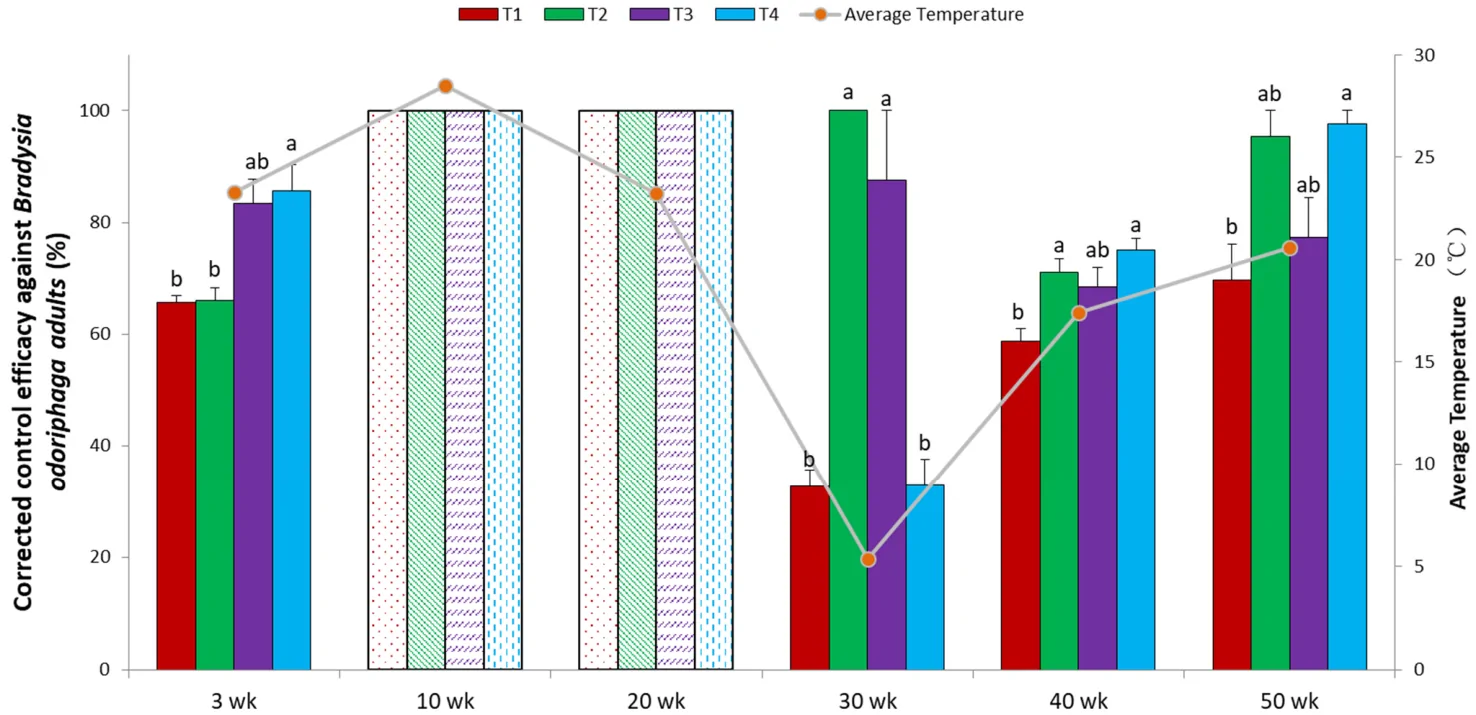
Corrected control efficacy against *Bradysia odoriphaga* adults under greenhouse conditions in different treatments. Values are mean ± SE. Different letters above columns indicate significant differences based on one-way ANOVA followed by Tukey’s HSD test; *p* < 0.05. CK, no control measure; T1, *Coenosia attenuata* alone; T2, *C. attenuata* + mulch layer; T3, *C. attenuata* + mulch layer + banker media system; T4, *C. attenuata* + banker media system. At weeks 10 and 20, high-temperature stress caused complete mortality of pests in both the control and all treatment groups. Corrected control efficacy could not be calculated. Raw mortality reached 100% at these two time points, which was not Abbott-corrected.

The corrected control efficacy against *B. odoriphaga* adults across different treatments was shown in Figure 2. Fungus gnat adults were suppressed by *C. attenuata* adults in T1 over the subsequent 50 weeks, with corrected control efficacy ranging from 32.68% at 30 weeks to 69.73% at 50 weeks. There was no significant difference in corrected control efficacy in T3 and T4, but both of them were significantly higher than that of T1 and T2 at 3 weeks (*F*_3,8_ = 6.97, *p* = 0.0127). Consistent with the observations for larvae, the 100% raw mortality of adults was also attributable to high temperature at 10 and 20 weeks.

The fungus gnat adults were suppressed by *C. attenuata* adults during the subsequent 50 weeks, with the corrected control efficacy of 65.94% at 3 weeks to 100% at 30 weeks in T2 (Figure 2). Especially at 30 weeks, the lowest temperature, T2 and T3, which utilized a mulch layer, exhibited significantly higher corrected control efficacy than T1 and T4 (*F*_3,8_ = 27.9, *p* = 0.0001). Compared with *B. odoriphaga* adults, adults of *C. attenuata* and *D. melanogaster* lost activity under low-temperature stress and possessed poor low-temperature tolerance. At 40 weeks, there was no significant difference in corrected control efficacy in T2 and T4, but both of them were significantly higher than that of T1 (*F*_3,8_ = 6.97, *p* = 0.0127). At 50 weeks, corrected control efficacy against *B. odoriphaga* adults did not differ significantly among treatments T2, T3, and T4. Nevertheless, T2 (mulch-layer treatment) and T4 (banker media treatment) exhibited significantly higher corrected control efficacy than T1 (*F*_3,8_ = 4.97, *p* = 0.031).

In treatment T3 with banker media and a mulch layer, *C. attenuata* adults suppressed fungus gnat adults over the subsequent 50 weeks, with corrected control efficacy ranging from 68.38% at 40 weeks to 87.61% at 30 weeks (Figure 2). Further, treatment T3 exhibited significantly higher corrected control efficacy than T1 and T2 at 3 weeks (*F*_3,8_ = 6.97, *p* = 0.0127). In treatment T4 containing the banker media system, *C. attenuata* adults suppressed fungus gnat adults across the subsequent 50-week period, with corrected control efficacy ranging from 32.83% at 30 weeks to 97.69% at 50 weeks (Figure 2). At 3, 40, and 50 weeks, treatment T4 exhibited significantly higher corrected control efficacy than treatment T1 (mulch-layer treatment) (*F*_3,8_ = 6.97, *p* = 0.0127; *F*_3,8_ = 6.97, *p* = 0.0127; *F*_3,8_ = 4.97, *p* = 0.031, respectively).

### 3.3. Plant Height of Chinese Chive in Greenhouse Conditions

**Figure 3:**
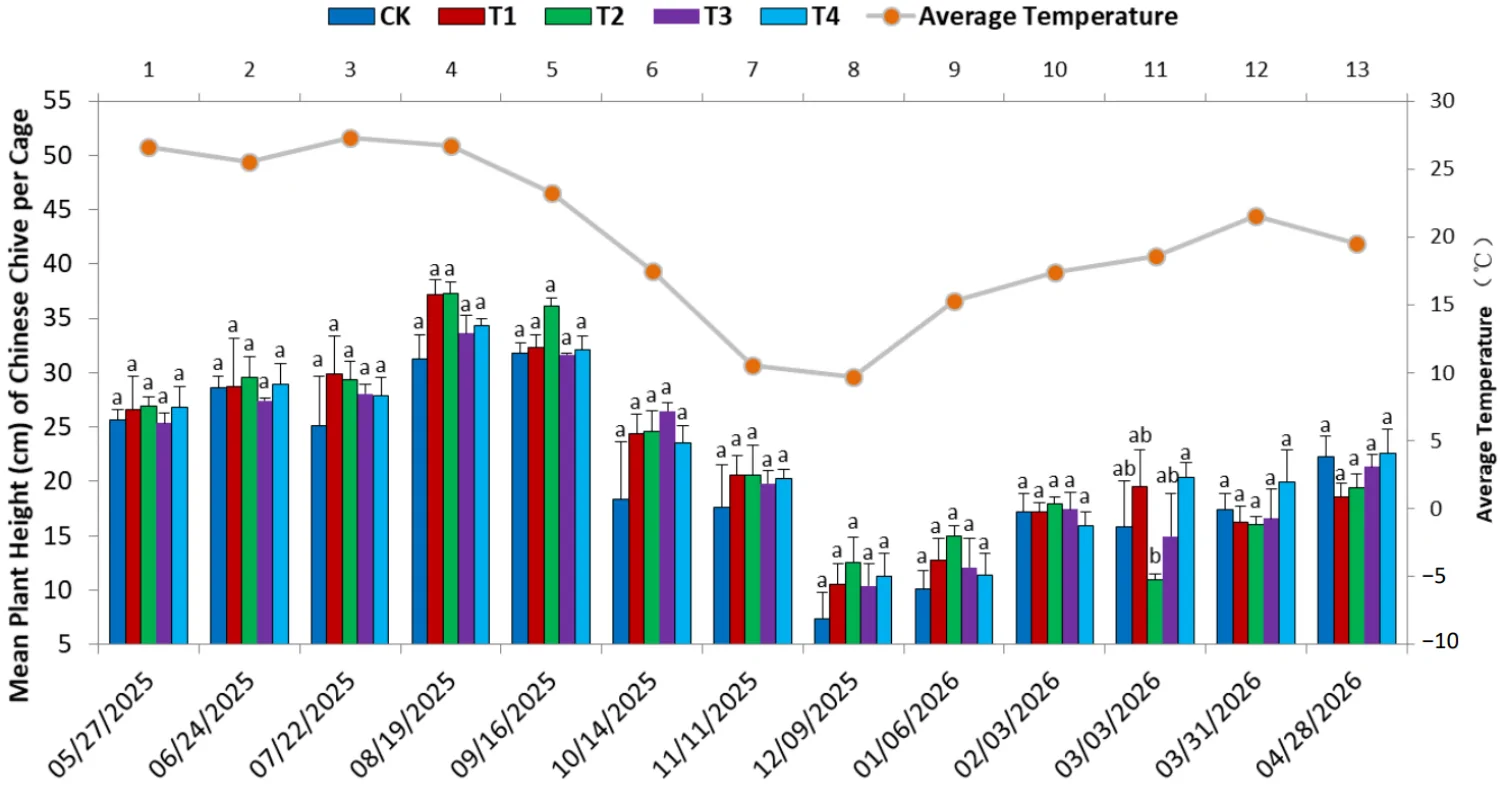
Mean plant height of Chinese chive under greenhouse conditions in different treatments. Values are means ± SE. Different lowercase letters above the bars indicate significant differences among treatments (α = 0.05). Levene’s test was used to assess homogeneity of variances. When variances were homogeneous, one-way ANOVA followed by Tukey’s HSD test was performed; when variances were heterogeneous, Welch’s ANOVA followed by the Games–Howell test was applied. CK, no control measure; T1, *Coenosia attenuata* alone; T2, *C. attenuata* + mulch layer; T3, *C. attenuata* + mulch layer + banker media system; T4, *C. attenuata* + banker media system.

Across all treatments, Chinese chive plant height increased under high-temperature conditions and decreased under low-temperature conditions. For CK, T1, T2, T3, and T4, plant height varied from 7.96 cm (9 December 2025) to 31.74 cm (16 September 2025), 10.51 cm (9 December 2025) to 37.18 cm (19 August 2025), 10.91 cm (3 March 2026) to 37.33 cm (19 August 2025), 10.39 cm (9 December 2025) to 33.63 cm (19 August 2025), and 11.21 cm (9 December 2025) to 34.34 cm (19 August 2025) (Figure 3).

No significant differences in Chinese chive plant height were detected among all treatments and CK on 27 May 2025, 24 June 2025, 22 July 2025, 19 August 2025, 16 September 2025, 14 October 2025, 11 November 2025, 9 December 2025, 6 January 2026, 3 February 2026, 31 March 2026, and 28 April 2026 (*F*_4,10_ = 0.17, *p* = 0.9497; Games–Howell test, all *p* > 0.05; *F*_4,10_ = 0.44, *p* = 0.7788; *F*_4,10_ = 3.05, *p* = 0.0697; Games–Howell test, all *p* > 0.05; *F*_4,10_ = 1.23, *p* = 0.3597; *F*_4,10_ = 0.27, *p* = 0.8885; *F*_4,10_ = 0.78, *p* = 0.563; *F*_4,10_ = 0.84, *p* = 0.532; *F*_4,10_ = 0.32, *p* = 0.8582; *F*_4,10_ = 0.6, *p* = 0.6731; *F*_4,10_ = 1.25, *p* = 0.3504, respectively). However, with the help of the banker media system, T4 exhibited significantly higher plant height on 3 March 2026 than T2 (Games–Howell test, *p* = 0.01) (Figure 3).

### 3.4. Leaf Chlorophyll Content of Chinese Chive in Greenhouse Conditions

**Figure 4:**
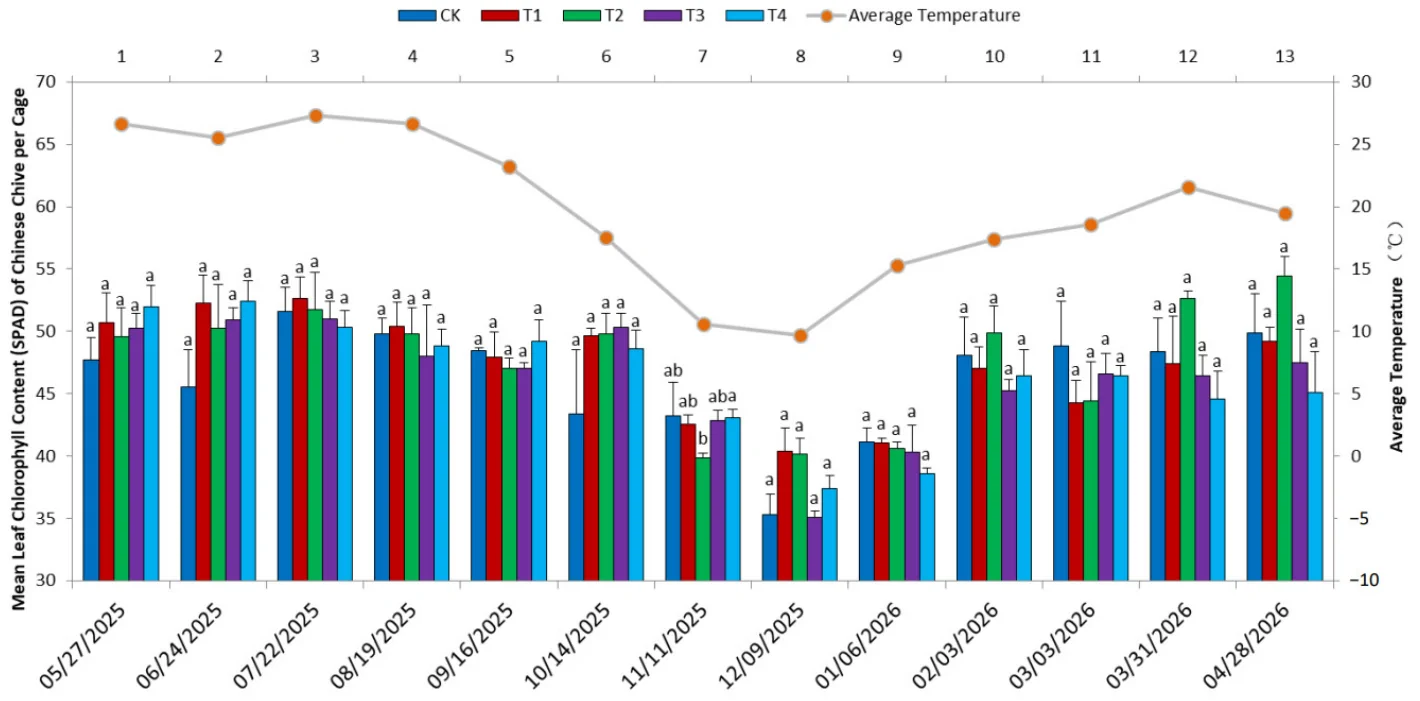
Mean leaf chlorophyll content of Chinese chive under greenhouse conditions in different treatments. Values are means ± SE. Different lowercase letters above the bars indicate significant differences among treatments (α = 0.05). Levene’s test was used to assess homogeneity of variances. When variances were homogeneous, one-way ANOVA followed by Tukey’s HSD test was performed; when variances were heterogeneous, Welch’s ANOVA followed by the Games–Howell test was applied. CK, no control measure; T1, *Coenosia attenuata* alone; T2, *C. attenuata* + mulch layer; T3, *C. attenuata* + mulch layer + banker media system; T4, *C. attenuata* + banker media system.

Consistent with the pattern observed for plant height, leaf chlorophyll content of Chinese chive increased under high-temperature conditions and decreased under low-temperature conditions across all treatments. For CK, T1, T2, T3, and T4, leaf chlorophyll content ranged from 35.26 SPAD (9 December 2025) to 51.6 SPAD (22 July 2025), 40.41 SPAD (9 December 2025) to 52.66 SPAD (22 July 2025), 39.85 SPAD (11 November 2025) to 54.46 SPAD (28 April 2026), 35.05 SPAD (9 December 2025) to 50.91 SPAD (24 June 2025), and 37.42 SPAD (9 December 2025) to 52.42 SPAD (24 June 2025) (Figure 4).

No significant differences in leaf chlorophyll content were detected among all treatments and CK on 27 May 2025, 24 June 2025, 22 July 2025, 19 August 2025, 16 September 2025, 14 October 2025, 9 December 2025, 6 January 2026, 3 February 2026, 3 March 2026, 31 March 2026, and 28 April 2026 (*F*_4,10_ = 0.66, *p* = 0.6338; *F*_4,10_ = 1.29, *p* = 0.3377; *F*_4,10_ = 0.19, *p* = 0.9356; *F*_4,10_ = 0.16, *p* = 0.9545; *F*_4,10_ = 0.58, *p* = 0.6839; Games–Howell test, all *p* > 0.05; *F*_4,10_ = 3.56, *p* = 0.0469, but no significant inter-group differences were detected by Tukey’s HSD multiple-comparison test; *F*_4,10_ = 0.81, *p* = 0.5469; *F*_4,10_ = 0.69, *p* = 0.6145; Games–Howell test, all *p* > 0.05; Games–Howell test, all *p* > 0.05; *F*_4,10_ = 1.92, *p* = 0.1841, respectively). However, with the help of the banker media system, T4 exhibited significantly higher leaf chlorophyll content on 11 November 2025 than T2 (Games–Howell test, *p* = 0.023) (Figure 4).

### 3.5. Dry Weight of Chinese Chive in Greenhouse Conditions

**Figure 5:**
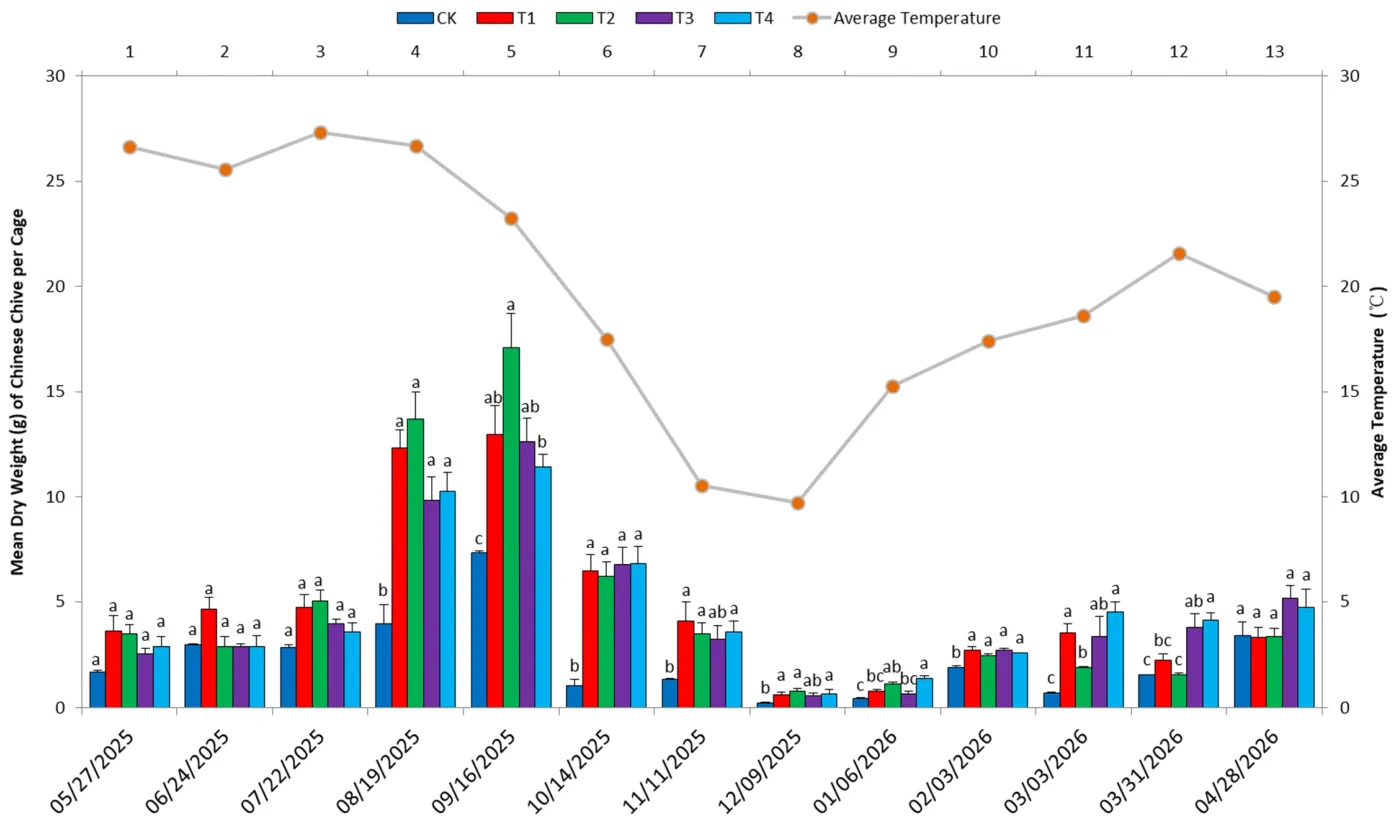
Mean dry weight of Chinese chive under greenhouse conditions in different treatments. Values are means ± SE. Different lowercase letters above the bars indicate significant differences among treatments (α = 0.05). Levene’s test was used to assess homogeneity of variances. When variances were homogeneous, one-way ANOVA followed by Tukey’s HSD test was performed; when variances were heterogeneous, Welch’s ANOVA followed by the Games–Howell test was applied. CK, no control measure; T1, *Coenosia attenuata* alone; T2, *C. attenuata* + mulch layer; T3, *C. attenuata* + mulch layer + banker media system; T4, *C. attenuata* + banker media system.

Across all treatments, Chinese chive dry weight fluctuated with mean temperature: it peaked in September and dropped to the minimum in December under the lowest mean temperature, followed by an upward increase from February as temperature rose (Figure 5). The dry-weight ranges for CK, T1, T2, T3, and T4 were 0.21–7.31 g, 0.63–12.97 g, 0.78–17.07 g, 0.57–12.61 g, and 0.65–11.44 g, corresponding to 9 December 2025 (minimum) and 16 September 2025 (maximum), respectively (Figure 5).

No significant differences in dry weight were detected among all treatments and CK on 27 May 2025, 24 June 2025, 22 July 2025, and 28 April 2026 (*F*_4,4.25_ = 6.35, *p* = 0.0567; *F*_4,4.334_ = 0.8425, *p* = 0.5578; *F*_4,4.56_ = 3.01, *p* = 0.1394; *F*_4,10_ = 1.91, *p* = 0.1852, respectively). CK exhibited significantly lower dry weight than T1, T2, T3, and T4 on 19 August 2025, 16 September 2025, 14 October 2025, 3 February 2026, and 3 March 2026 (*F*_4,10_ = 12.67, *p* = 0.0006; Games–Howell test, all *p* ≤ 0.0499; *F*_4,10_ = 11.65, *p* = 0.0009; Games–Howell test, all *p* ≤ 0.048; Games–Howell test, all *p* ≤ 0.048, respectively).

Treatment T2 had a numerically higher dry weight than T1 on 19 August 2025 and 16 September 2025, although no significant difference was detected between the two treatments (Tukey’s HSD test, adjusted *p* = 0.7915; Games–Howell test, *p* = 0.2627, respectively). By contrast, T2 showed a significantly lower dry weight than T1 on 3 March 2026 (Games–Howell test, *p* = 0.04).

T3 exhibited numerically greater dry weight than T2 on 3 March 2026, although no significant difference was observed between the two treatments (Games–Howell test, *p* = 0.557). Likewise, dry weight did not differ significantly between T3 and T4 on 3 March 2026 (Games–Howell test, *p* = 0.793). Both T3 and T4 had significantly greater dry weights than T2 (Games–Howell test, *p* = 0.045 and 0.004, respectively), whereas no significant difference was found between T3 and T4 on 31 March 2026 (Games–Howell test, *p* = 0.987).

Across the one-year experiment, the total mean cumulative dry weight of Chinese chive per cage was 29.28 g (CK), 62.09 g (T1), 63.03 g (T2), 58.00 g (T3), and 59.40 g (T4). The cumulative dry weights of Chinese chives under the four treatments T1, T2, T3, and T4 were all significantly higher than those of the control group CK (*F*_4,10_ = 7.57, *p* = 0.0045), increasing by 112.06%, 115.27%, 98.09%, and 102.87%, respectively, compared with the cumulative dry weight of CK. Yield of Chinese chive was enhanced in T1, T2, T3, and T4 following the release of *C. attenuata*, compared with the CK treatment without *C. attenuata*.

## 4. Discussion

**Figure 6:**
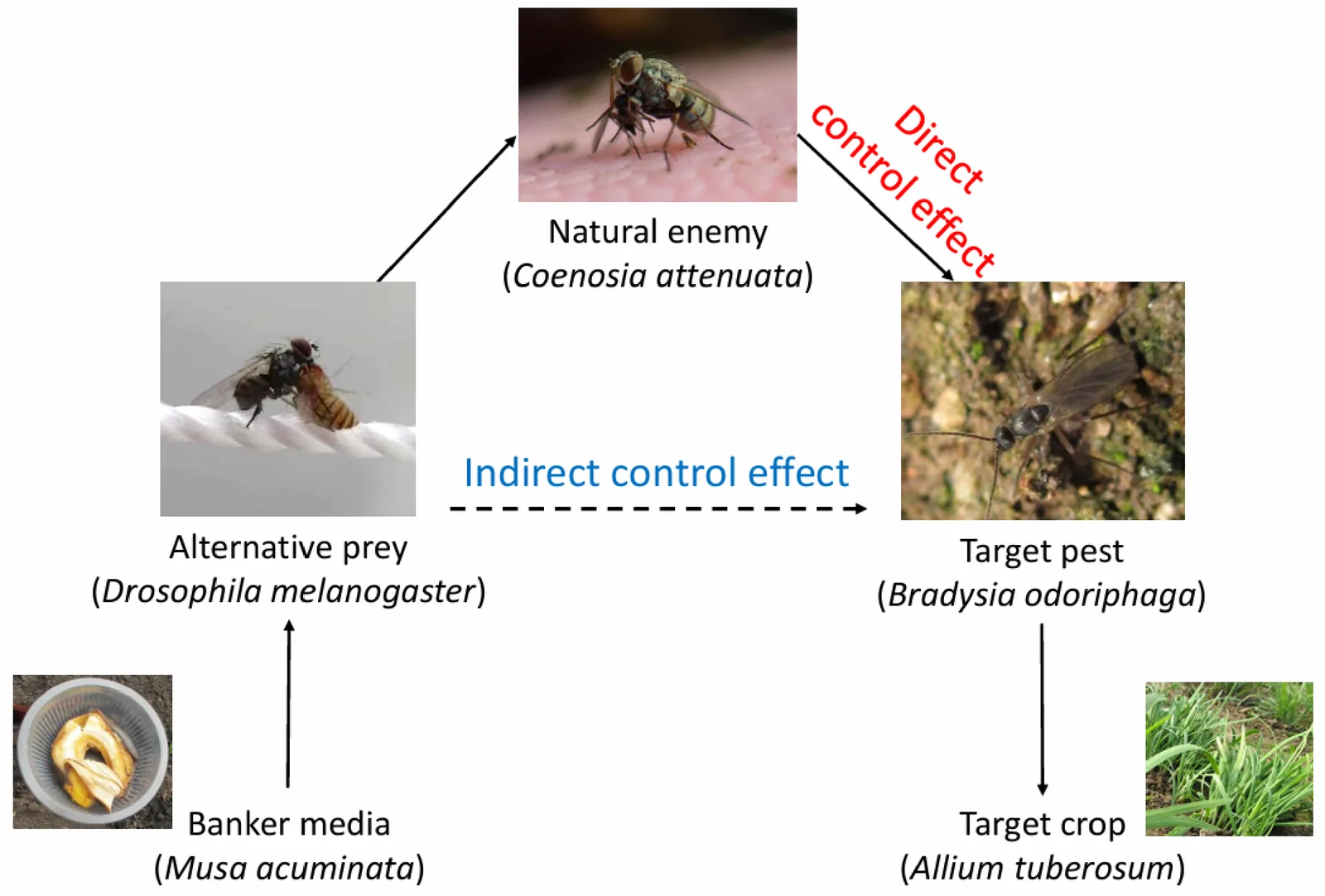
Banker media system for biocontrol of *Bradysia odoriphaga* using *Coenosia attenuata*.

At 25 °C, *B. odoriphaga* adults live only 3.62–4.39 days [37], while *C. attenuata* adults survive approximately 20 days longer. To ensure that adult *C. attenuata* have sufficient food when the *B. odoriphaga* adults decline or are absent, we introduced a banker media system to enhance biocontrol efficacy in Chinese chive fields (Figure 6). This system may prolong *C. attenuata* population persistence, increasing predation on *B. odoriphaga* adults. Consequently, the combined strategy nearly doubled the dry weight of *A. tuberosum* under greenhouse conditions, a value approaching that of plants treated with entomopathogenic nematodes and imidacloprid [38].

In vitro, 100% mortality occurred when adults, eggs, larvae, or pupae were exposed to a constant 40 °C for 1.3, 1.8, 2.8, or 3.7 h, respectively [12], and *B. odoriphaga* adults die before oviposition under high summer temperatures (>30 °C) [39,40,41]. Consistently, in this study, field *B. odoriphaga* populations nearly disappeared between July and September, when mean temperatures remained above 22 °C, resulting in 100% raw mortality at 10 and 20 weeks in all treatments. We therefore infer that this 100% raw mortality was largely attributable to high-temperature stress. In greenhouses, approximately 4 h of daily temperatures exceeding 30 °C around noon in summer can cause *B. odoriphaga* populations to collapse. Thus, in addition to natural enemies such as predatory flies, high temperature itself can effectively suppress *B. odoriphaga*; under such high-temperature conditions, releasing natural enemies at low density may still achieve effective control.

*Coenosia attenuata* is active across a broad temperature range (≈12 to 42 °C) [42]. Our field observations showed that the activity intensity of adult *C. attenuata* was significantly reduced under sustained mean temperatures below 15 °C from mid-November to early January. However, as temperatures rise during the day, both *C. attenuata* and *B. odoriphaga* activity can recover to a certain level, making control possible. At a mean temperature of 15 °C, the control efficacy of *C. attenuata* releases may be limited, and this temperature is not recommended for release; this requires further verification that accounts for the actual temperature fluctuation patterns in the greenhouse.

Maintaining *C. attenuata* adult populations by provision of *Drosophila* adults and banker media is a promising strategy to improve the biocontrol efficacy of *C. attenuata* against *B. odoriphaga*. This was supported by our present findings, in which treatment T4 achieved higher corrected control efficacy against both *B. odoriphaga* larvae and adults compared to T1 (which used only *C. attenuata*). Although *C. attenuata* adults prefer fungus gnats over other prey [43], the release density of *Drosophila* adults should not be excessively high. We hypothesize that excess alternative prey may compete with the target pest, potentially reducing predation pressure on *B. odoriphaga*. Furthermore, the flight of *C. attenuata* individuals was increased in response to increases in the number of prey flights [44]. Our laboratory observations indicated that frequent flight activity of *C. attenuata* may cause wing abrasion and shorten adult longevity [33].

Mulch layer is an important habitat modification that benefits the development and conservation of naturally occurring biological agents. Grosman et al. 2011 [45] found that mulch layers used for chrysanthemum support the establishment of astigmatic mites and, as a result, increase densities of soil-dwelling predatory mites [45]. Similar methods have been reported to support the generalist hunter fly *C. attenuata* with the help of a wood fiber substrate [22]. Martins et al. (2015) found that the addition of coir substrate to a soil substrate improved the rearing efficiency of *C. attenuata* [46]. In addition, coir substrate could be used as an oviposition substrate and rearing substrate in some experiments [22,27,47]. In our study, coir substrate was successful as a mulch layer to enhance the biological control of *B. odoriphaga* with *C. attenuata*. Additionally, Figueiredo et al. 2012 [48] found that mucus produced by earthworms induced *C. attenuata* females to extend their ovipositors and lay more eggs in some situations. They concluded that mucus acts as a kairomone. Hence, developing mulch layers with kairomone for supporting *C. attenuata* in greenhouse crops seems to be a promising method. In treatment T3, the combined application of the banker media system and mulch layer did not generate synergistic effects. The 1.5 m^3^ volume of the experimental cage may be a plausible contributing factor.

Plant height and leaf chlorophyll content of Chinese chive were not significantly affected by feeding damage from *B. odoriphaga* larvae throughout the experiment. Therefore, plant height and leaf chlorophyll content are not suitable assessment parameters for evaluating whether the underground roots and stems of Chinese chive sustained damage from *B. odoriphaga* larvae.

The banker media system and/or mulch layer also hold promise for future testing as an enhancement measure for biocontrol of whiteflies, leafminers, and spotted-wing drosophila (*Drosophila suzukii*) using *C. attenuata*. Nevertheless, *C. attenuata* larvae are unable to feed on above-ground stages, including whitefly nymphs, leafminer larvae, or *D. suzukii* larvae. When releasing *C. attenuata* adults for suppressing adult target pests, a banker media system consisting of kidney bean powder, black peat, coir substrate, and fungus gnat eggs and/or larvae can be deployed in greenhouses, open fields, and orchards. *C. attenuata* adults can oviposit within the banker media, and newly hatched larvae subsequently prey upon fungus gnat larvae in banker media. Banker media containing *C. attenuata* eggs and/or larvae can then be transferred to the insectary for rearing; newly emerged adults are thereafter collected, transported, and released at target sites. Collectively, our results highlight that banker media systems containing crop-safe alternative prey, or a mulch layer combined with kairomones to stimulate predator oviposition, deserve further research.

## 5. Conclusions

In conclusion, the predatory tiger fly *C. attenuata* effectively suppressed the Chinese chive maggot *B. odoriphaga* on *A. tuberosum*, with its efficacy markedly enhanced by a banker media system or a coir mulch layer, which also increased chive yield. These findings identify both measures as effective auxiliary tools for *C. attenuata*-based biocontrol in protected cultivation and support integrated pest management strategies that reduce reliance on chemical insecticides.

## Figures and Tables

**Table 1:** Five different treatments in this experiment.

Treatments	Chinese Chive Maggot ^a^	Tiger Fly ^b^	Mulch Layer ^c^	Banker Media System ^d^
CK	*Bradysia odoriphaga*	---	---	---
T1	*B. odoriphaga*	*Coenosia attenuata*	---	---
T2	*B. odoriphaga*	*C. attenuata*	coir substrate	---
T3	*B. odoriphaga*	*C. attenuata*	coir substrate	bananas and *D. melanogaster*
T4	*B. odoriphaga*	*C. attenuata*	---	bananas and *D. melanogaster*

^a^ Fifteen and 30 pairs of newly emerged *B. odoriphaga* adults were released in each cage on 26 March 2025 and 3 April 2025, respectively. ^b^ One male and 2 females of newly emerged *C. attenuata* adults were first released in each cage on 29 April 2025. Then 1 female and 1 male, 4 females and 2 males, 3 females and 1 male, 2 females and 2 males, 1 female and 1 male, 2 females and 1 male newly emerged *C. attenuata* adults were intermittently released in each cage on 13 May 2025, 7 October 2025, 16 December 2025, 17 February 2026, 24 February 2026, and 6 April 2026, respectively. ^c^ One-centimeter-thick layer of moist coir was added on the soil surface of the flowerpot on 29 April 2025. The moist coir was put on the soil surface of the flowerpot again every week after investigation. ^d^ Twenty pairs of newly emerged *D. melanogaster* adults, reared with banana in the laboratory, were first released in each cage on 30 March 2025. One banana was put in a plastic pot (15 cm top diameter, 8 cm bottom diameter, and 6 cm high) surrounded by flowerpots in the cage and periodically replenished. Then 20, 20, 15, 20, 10, 25, 5 pairs of newly emerged *D. melanogaster* adults were released in each cage on 3 April 2025, 21 May 2025, 25 June 2025, 10 July 2025, 9 September 2025, 24 September 2025, and 9 October 2025, respectively.

## Data Availability

The minimal dataset necessary to support the conclusions of this study has been uploaded in the Data Availability Statements module in the submission system. The full raw dataset includes a large amount of unpublished sequential field-sampling data reserved for follow-up studies of our research group and cannot be fully publicly released. The supporting minimal dataset can be provided by the authors upon reasonable request.
